# Inflammatory Response in COVID-19 Patients Resulting from the Interaction of the Inflammasome and SARS-CoV-2

**DOI:** 10.3390/ijms22157914

**Published:** 2021-07-24

**Authors:** So Yeong Cheon, Bon-Nyeo Koo

**Affiliations:** 1 Department of Biotechnology, College of Biomedical & Health Science, Konkuk University, Chungju 27478, Korea; 2 Department of Anesthesiology and Pain Medicine, Yonsei University College of Medicine, Seoul 03722, Korea; 3 Anesthesia and Pain Research Institute, Yonsei University College of Medicine, Seoul 03722, Korea

**Keywords:** coronavirus disease 2019, severe acute respiratory syndrome coronavirus 2, inflammasome, cytokines

## Abstract

The outbreak of the coronavirus disease 2019 (COVID-19) began at the end of 2019. COVID-19 is caused by infection with the severe acute respiratory syndrome coronavirus 2 (SARS-CoV-2) and patients with COVID-19 may exhibit poor clinical outcomes. Some patients with severe COVID-19 experience cytokine release syndrome (CRS) or a cytokine storm—elevated levels of hyperactivated immune cells—and circulating pro-inflammatory cytokines, including interleukin (IL)-1β and IL-18. This severe inflammatory response can lead to organ damage/failure and even death. The inflammasome is an intracellular immune complex that is responsible for the secretion of IL-1β and IL-18 in various human diseases. Recently, there has been a growing number of studies revealing a link between the inflammasome and COVID-19. Therefore, this article summarizes the current literature regarding the inflammasome complex and COVID-19.

## 1. Introduction

The coronavirus disease 2019 (COVID-19) is caused by severe acute respiratory syndrome coronavirus 2 (SARS-CoV-2) infection [1]. Some individuals infected with SARS-CoV-2 have no symptoms, while some experience severe fever, pneumonia, and even acute respiratory distress syndrome (ARDS) [1]. SARS-CoV-2, which is transmitted via respiratory droplets, replicates in the lower respiratory tract and aggravates the inflammatory response, thereby inducing pneumonia and damage in the airway [2]. Some patients with COVID-19 exhibit immune disturbances such as cytokine release syndrome (CRS) and macrophage activation syndrome (MAS) [3,4]. Hyper-cytokinemia, i.e., the increase in pro-inflammatory and anti-inflammatory cytokine levels, occurs in severe cases. CRS or the ‘cytokine storm’ contributes to organ damage/failure and death [3,4]. This cytokine storm was recently shown to be possibly caused by the S1 subunit of the SARS-CoV-2 spike (S) protein [5]. Furthermore, COVID-19 patients with severe respiratory failure show MAS or immune dysregulation, characterised by hyperactivated monocytes, CD4^+^ lymphopenia, B lymphopenia, and natural killer (NK) cytopenia [3]. COVID-19 patients are also reported to show CD4^+^ lymphopenia; CD8^+^ lymphopenia; and higher levels of cytokines such as interleukin (IL)-6, IL-10, and tumor necrosis factor alpha (TNF-α) [6]. Based on these studies, serious immunological features of patients with COVID-19 have been reported.

There have been a growing number of studies on inflammasome activation induced by SARS-CoV-2 infection and COVID-19 severity in patients [2,7]. In particular, the SARS-CoV-2 S protein primes and drives the activation of the nucleotide-binding oligomerisation domain (NOD)-like receptor family pyrin domain containing 3 (NLRP3) inflammasome, and the SARS-CoV-2 S1 protein triggers inflammasome activity alone [8,9]. The inflammasome is a macromolecular immune complex in cells, and it is involved in the extracellular release of IL-1β and IL-18 as well as in pyroptotic cell death [10]. Aberrant inflammasome activation is related to the pathogenesis or pathophysiology of various diseases such as autoimmune diseases, infectious diseases, and neurodegenerative diseases [11]. More importantly, these abnormally activated inflammasomes and inflammasome-driven cytokines are observed in patients with COVID-19. Therefore, in this review, we focused on the inflammatory immune response in patients with COVID-19 through the interaction between the inflammasome and SARS-CoV-2.

## 2. Inflammasome Complex during Viral Infection

**Figure 1:**
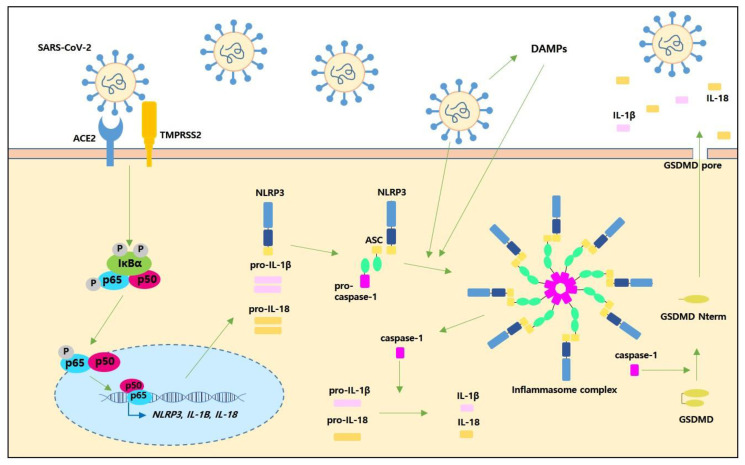
Possible routes of SARS-CoV-2 to activate the inflammasome complex. SARS-CoV-2 enters the cell via the angiotensin-converting enzyme II (ACE2) and transmembrane serine protease 2 (TMPRSS2). SARS-CoV-2 itself may activate the nuclear factor kappa-light-chain enhancer of activated B cells (NF-κB) signalling pathway, and NF-κB subunits are involved in the expressions of *NLRP3*, *IL-1B*, and *IL-18*. SARS-CoV-2 viroporin might activate the NLRP3 inflammasome complex. The NLRP3 inflammasome assembles with the adaptor protein ASC and pro-caspase-1. Inflammasome activation is responsible for the cleavage of caspase-1 and the promotion of IL-1β and IL-18 secretion. Cleaved caspase-1 results in the cleavage of gasdermin D (GSDMD). Cleaved GSDMD creates transmembrane pores, and IL-1β and IL-18 are secreted into the extracellular environment, ultimately leading to pyroptosis.

Multiple internal or external stimuli, including bacterial and viral infection, can activate the intracellular immune complex called the inflammasome, which is comprised of pattern recognition receptors (PRRs) such as absent in melanoma 2 (AIM2)-like receptor (ALR) and NLR, the adaptor protein apoptosis-associated speck-like protein containing a CARD (ASC), and pro-caspase-1 [10]. In particular, upon viral infection, ALR or NLR families, including NLRP1, NLRP2, NLRP3, and AIM2, detect pathogen-associated molecular patterns (PAMPs) and assemble an intracellular inflammasome complex by recruiting ASC and pro-caspase-1 [12,13]. This innate immune complex exerts antiviral properties by eradicating invading viruses, a process that plays a pivotal role in the host defense system [10]. In the case of the NLRP3 inflammasome complex, two steps are required for priming and activation. The priming step is triggered by the activation of receptors due to viral infection, including toll-like receptors (TLRs) and RIG-I-like receptors (RLRs). The viral infection initiates the intracellular nuclear factor kappa-light-chain enhancer of the activated B cells (NF-κB) signalling pathway, which contributes to *NLRP3*, *IL-1B*, and *IL-18* expressions [10,14]. The activation step is initiated by various intra/extracellular stimuli, ranging from adenosine triphosphate (ATP), reactive oxygen species (ROS), lysosomal destabilisation, invading pathogens, viral infection-induced alteration in plasma membrane integrity, and ion fluxes [10,14]. The NLRP3 inflammasome assembles with ASC and pro-caspase-1, thereby leading to the cleavage of caspase-1. This active cleaved caspase-1 promotes IL-1β and IL-18 maturation and the cleavage of gasdermin D (GSDMD) [15]. Cleaved GSDMD (GSDMD ^Nterm^) forms transmembrane pores that allow IL-1β and IL-18 to be secreted into the extracellular environment, which leads to pyroptosis, a lytic type of cell death [16] (Figure 1). Activation of canonical inflammasome and caspase-1, and the non-canonical inflammasome and caspase-11 (caspase-4/5 in humans) are responsible for pyroptosis [14]. In non-canonical inflammasome activation, caspase-11 responds to intracellular lipopolysaccharide (LPS) from Gram-negative bacteria; LPS in the cytosol binds to the CARD motif of caspase-11, resulting in the oligomerisation of caspase-11 that activates the non-canonical inflammasome [17]. Caspase-11 is involved in the cleavage of GSDMD, and caspase-11-cleaved GSDMD triggers the formation of membrane pores, which causes potassium efflux, further leading to NLRP3 inflammasome-related caspase-1 activation [18]. Under infectious conditions, inflammasome complex activation leads to pyroptosis of infected cells that secrete damage-associated molecular patterns (DAMPs), thereby leading to inflammatory responses [13]. However, abnormal activation or dysregulation of the inflammasome complex can exacerbate the host condition, resulting in pathological injury such as that caused by increased levels of inflammatory cytokines and cellular and tissue damage [14,15].

## 3. Inflammasome Complex and SARS-CoV-2 Infection in COVID-19 Patients

The SARS-CoV-2 S protein uses the angiotensin-converting enzyme 2 (ACE2) receptor as an entry receptor and transmembrane serine protease 2 (TMPRSS2) to cleave the S-protein in order to gain entry into the host cells [19]. SARS-CoV-2 infection is implicated in the activation of NF-κB signalling, which is known to initiate the transcription of a variety of genes such as TNF-α, IL-1β, IL-6, IL-8, and monocyte chemoattractant protein-1 (MCP-1), which are responsible for innate and adaptive immune responses [20]. In addition, the SARS-CoV-2 S1 protein is involved in the production of IL-1β, IL-6, IL-8, and TNF-α in human peripheral blood mononuclear cells (PBMCs); the S1 protein also triggers NF-κB signalling by phosphorylation of p65 and IκBα and the degradation of IκBα [9]. Similarly, the SARS-CoV-2 S1 protein alone has been related to the increased levels of NF-κB and IL-1β, and it provokes the cytokine storm by eliciting TNF-α, IL-1β, IL-6, and IL-17 in vivo [5]. Moreover, the S1 protein enhances the DNA binding of the NF-κB p65 subunit in the nucleus [9] (Figure 1). In the case of SARS-CoV, analogous to the SARS-CoV-2, the open reading frame 8b (ORF8b) forms insoluble aggregates and is involved in the activation of the NLRP3 inflammasome [21]. In addition, SARS-CoV viroporin 3a promotes the activation of the NLRP3 inflammasome and the secretion of IL-1β [22]. The interplay between ACE2 and SARS-CoV-2 also triggers the NLRP3 inflammasome [23]. The SARS-CoV-2 S protein was recently shown to prime inflammasome formation and drive NLRP3 inflammasome activation in macrophages derived from patients with COVID-19 [8]. The SARS-CoV-2 S1 protein increases the NLRP3 level and caspase-1 activity in human PBMCs [9]. Uncontrolled responses of monocytes and macrophages to SARS-CoV-2 infection may be the key source of the hyper-inflammatory response in the circulation [24,25]. Interestingly, SARS-CoV-2 RNA provokes an inflammatory response more than that caused by SARS-CoV-1 RNA [26]. Intensive care unit (ICU) patients in Wuhan, China who contracted pneumonia after SARS-CoV-2 infection showed high levels of cytokines in their plasma, including IL-2, IL-7, TNF-α, and MCP-1 [27]. Recent studies have demonstrated the potential role of the inflammasome complex during the COVID-19 cytokine storm [7,28], and the amount of clinical evidence showing a link between the inflammasome complex and COVID-19 is increasing. A recent study reported the direct involvement of the inflammasome complex in COVID-19 [7]. Patients with COVID-19 show high concentrations of active/cleaved caspase-1 and IL-18 in their sera. Concomitantly, the levels of cytokines such as IL-4, IL-6, and IL-10 are also increased in the sera of patients with COVID-19 compared with those of controls. PBMCs from patients with COVID-19 exhibit active intracellular caspase-1, increased expressions of NLRP3 and ASC, and the secretion of IL-1β [7]. Furthermore, primary human monocytes after infection with SARS-CoV-2 exhibit caspase-1 activation and IL-1β production by inducing NLRP3 inflammasome activation [7]. SARS-CoV-2-infected cells undergo pyroptosis and secrete DAMPs, including ASC oligomers [2]. In addition, SARS-CoV-2 infection has caused pyroptosis-induced lytic cell death in human primary monocytes, a process that may contribute to the exacerbated inflammatory response and leukocytopenia observed in patients with severe COVIID-19 [28]. In particular, SARS-CoV-2 infection triggers the inflammasome complex in monocytes, inducing the cleavage of caspase-1, IL-1β production, and the cleavage of GSDMD, an executioner of pyroptosis [28]. Clinically, circulating monocytes isolated from patients with severe COVID-19 exhibit increased caspase-1 activation and higher levels of lytic cell death compared with monocytes from healthy controls [28]. Higher IL-1β levels are observed in the plasma of severely ill patients with COVID-19 [28]. The SARS-CoV-2 S protein promotes NLRP3 inflammasome activation and IL-1β secretion in COVID-19 patient-derived macrophages, and stimulation of the S protein upregulates both IL-1β and TNF-α; however, TNF-α is secreted in an NLRP3 inflammasome-independent manner [8]. In RNA sequencing, the SARS-CoV-2 S protein is observed to upregulate innate immunity-associated genes and several signalling pathways, including pro-inflammatory pathways, the NOD-like receptor, and the inflammasome signalling pathway. In particular, the S protein increases TLR2 in CD14^+^ macrophages, and the S protein is correlated with a high level of TLR2 in cells derived from patients with COVID-19 [8]. In a single-cell RNA sequencing analysis, the upregulation of caspase expression in immune cells is observed in blood from patients with COVID-19. In particular, caspase-1 expression is distinct in CD4^+^ T lymphocytes in hospitalized patients with COVID-19. In addition, an increased IL-18 level is found in the sera of hospitalized patients with COVID-19 [29]. An epigenome-wide association study showed that DNA methylation participates in the immune response to COVID-19, and the DNA methylation of 44 CpG sites were associated with inflammasomes, interleukins, cytokines, and the major histocompatibility complex (MHC), which are all relevant to COVID-19 severity [30]. In particular, epigenetic loci are found in the *AIM2* and MHC class I, C (*HLA-C*) genes [30]. Consistent with these findings, the levels of inflammasome-associated cytokines, including IL-1β and IL-18, were increased in the sera of patients with COVID-19 in the ICU [31]. Therefore, the NLRP3 inflammasome in myeloid cells has been proposed as a biomarker of COVID-19 severity [31].

Previous reports have suggested this relationship with the inflammasome complex not only in immune cells but also in other types of cells [32,33]. SARS-CoV-2 infection upregulates the genes involved in innate immunity and inflammasomes in human induced pluripotent stem cell (iPSC)-derived lung organoids (LORGs). In the LORGs, inflammasome-associated genes, including *NLRC4*, *NLRP3*, *ASC*, *CASP1* (caspase-1), and *IL-18*, and innate immunity-associated genes such as *TLR3* and *TLR7*, are upregulated in response to SARS-CoV-2 infection. The altered expression of these genes may be responsible for pulmonary inflammation and cell damage/death in the lungs [32]. Damaged hematopoietic stem and progenitor cells (HSPCs) and endothelial progenitor cells (EPCs) are found in patients with severe COVID-19 [33]. More specifically, ACE2 and TLR4 are highly observed on the extracellular membrane surfaces of HSPCs and EPCs. Stimulation of the SARS-CoV-2 S protein induces NLRP3 inflammasome activation and cytosolic activity of caspase-1 in human CD34^+^ HSPCs and CD34^+^ CD133^+^ CD31^+^ CD144^+^ EPCs. Moreover, the level of released lactate dehydrogenase (LDH) is increased in CD34^+^ cells; however, blocking the interaction between the S protein and ACE2 by recombinant human ACE2 results in the suppression of the NLRP3 inflammasome and caspase-1 activity. Treatment with TLR4 inhibitor TAC-242 and MCC950 also exhibits the same effects [33].

## 4. Failure of the Adaptive Immune Response by Aberrant Inflammasomes in Response to SARS-CoV-2

Patients with COVID-19 display lymphopenia (CD4^+^ and CD8^+^ T lymphocyte deficiency) and reduced interferon (IFN)-γ expression in CD4^+^ T lymphocytes, which are responsible for the severity of COVID-19 [34]. It is also reported that most patients with COVID-19 exhibit some form of immune deficiency, including lymphopenia in 83.2% of patients, thrombopenia in 36.2% of patients, and leukopenia in 33.7% of patients [35]. Patients with severe COVID-19 show markedly reduced CD4^+^ and CD8^+^ T lymphocyte levels compared to patients with moderate COVID-19, and the blood of patients with both severe or moderate COVID-19 is deficient in CD4^+^ CD25^+^ CD127^lo^ and CD45RA^+^ regulatory T lymphocytes [34]. Some patients with severe or moderate COVID-19 show a deficient expression of IFN-γ in CD4^+^ T lymphocytes, CD8^+^ T lymphocytes, and NK cells; however, lower levels of IFN-γ expression by CD4^+^ T lymphocytes are found in those with severe rather than moderate COVID-19 [34]. Thus, a decreased amount of circulating T lymphocytes is consistent with the reduced expression of IFN-γ. In particular, CD3^+^ CD4^+^ helper T (Th) cells and CD3^+^ CD8^+^ suppressor T cells were detected below normal levels in patients with COVID-19, and CD3^+^ CD4^+^ CD45RO^+^ memory Th cells and CD3^+^ CD8^+^ CD28^+^ cytotoxic suppressor T cells were found to be decreased in severe COVID-19 patients as compared to non-severe patients in Wuhan, China [36]. These findings support that COVID-19 recovery is implicated in memory formation in T cells [37]. Inflammasome activation leads to immune disturbance and lymphopenia, which are linked to adaptive immune system failure [38]. Inflammasome activation is responsible for pyroptotic T cell death, which may drive T-cell lymphopenia [38]. In one clinical study, high activity of caspase-1 was detected in CD3^+^ CD4^+^ CD45^+^ T cells and CD3^+^ CD45^+^ T cells, and increased levels of lactate dehydrogenase, a marker of pyroptosis, and IL-18 were observed in COVID-19 patients. In the lethal course of COVID-19, individuals with T-cell lymphopenia have been found to be more susceptible to pathological outcomes [38]. Inflammasome-induced pyroptosis of T cells results in the suppression of the adaptive immune system and the induction of the inflammatory response. In addition, abnormal distribution and dysregulated innate immune cells have an effect on the adaptive immune response. For instance, circulating myeloid cells show distinct phenotypes, i.e., a reduction of non-classical monocytes [39]. In COVID-19, an aberrant monocyte compartment is observed in a time- and severity-dependent manner [40]. All patients with COVID-19 exhibited a marked reduction of the CD14^lo^ CD16^hi^ non-classical monocyte. Patients with mild COVID-19 show increased levels of HLA-DR^hi^ CD11c^hi^/HLA-DR^hi^ CD83^hi^ inflammatory monocytes. Additionally, dysfunctional mature neutrophils and HLA-DR^lo^ monocytes are observed in patients with severe COVID-19 [40]. Taken together, the abnormal distribution and number of immune cells as well as the dysregulated communication between innate and adaptive immune cells due to aberrant inflammasomes and insufficient/deficient lymphocytes caused by pyroptotic cell death may contribute to an abnormal adaptive immune response.

## 5. Involvement of Inflammasomes in SARS-CoV-2-Derived Organ Damage in Patients with COVID-19 and Comorbidities

The cytokine storm is implicated in the tissue injury observed in patients with severe COVID-19 [41]. SARS-CoV-2 can damage many organs in the body, including the lungs, liver, kidneys, and heart as well as the blood, resulting in death due to multiple organ failure [42]. Furthermore, the presence of comorbidities in patients with COVID-19 is implicated in poor clinical outcomes, with these patients having a higher risk of mortality [43,44]. Many patients with COVID-19 have at least one underlying disease such as diabetes, hypertension, cerebrovascular disease, chronic renal diseases, or coronary heart disease [35]. Recently, autopsies of children and adolescents with COVID-19 revealed that SARS-CoV-2 had infected their lungs, kidneys, and hearts [45]. The patients exhibited pulmonary diseases with severe acute respiratory disease or multi-system inflammatory syndrome [45].

Damages to organs including the brain, kidney, muscle, and spleen—acute tubular necrosis and congestion in the kidney; splenitis, haemorrhages, and lymphoid hypoplasia in the spleen; reactive microglia, neuronal ischemia, and congestion in the brain; and myolysis and necrotic fibers in the muscles—were observed in patients with COVID-19 [45]. From the COVID-19 autopsies, marked vascular changes were observed in the blood vessels, which are known to express ACE2 on the surface of endothelial cells in the vascular system [46]. The blood vessels had severe phenotypes such as pulmonary arterial thromboemboli, deep vein thrombosis, and hypercoagulability [46]. The autopsies of 23 patients with COVID-19 showed severe capillary fibrin deposition, micro-haemorrhage, and capillary dilation in the myocardium [47]. These patients had comorbidities, including hypertension, coronary artery diseases, and diabetes mellitus [47]. In some cases, elevated interstitial CD68^+^ macrophage numbers were reported [47]. Moreover, patients with COVID-19 showed distinct hepatic symptoms [48]. Aberrant hepatic enzymes, hyperplasia, platelet-fibrin microthrombi, lobular inflammation, ischaemic hepatic necrosis, and steatosis were observed in the post-mortem livers of patients with COVID-19 [48]. One of the major causes of death of patients with COVID-19 was respiratory failure [49]. In particular, interstitial fibrosis and diffuse alveolar damage due to the disruption of the blood–air barrier and the infiltration of immune cells in the lungs were found in the autopsies of patients with COVID-19 [49]. Among 26 COVID-19 cases, 20 had pre-existing diseases such as chronic cardiovascular diseases (38.5%), hypertension (34.6%), and chronic pulmonary diseases (23.1%) [49]. Post-mortem lung tissues from patients with COVID-19 exhibit enormously increased numbers of NLRP3 and ASC-positive cells compared to those of control lung tissues [7]. Similarly, inflammasome components, including NLRP3, ASC specks, and caspase-1, are found in leukocytes of post-mortem lung samples of patients with fatal cases of COVID-19 [50]. In addition, elevated macrophage infiltration and ASC speck formation are observed in post-mortem lungs of patients with COVID-19 [51]. Patients with COVID-19 develop a highly severe fibrotic response in the lung, and patients with idiopathic pulmonary fibrosis (IPF) are at high risk of severe COVID-19 [52]. Interestingly, NLRP3 inflammasome is directly involved in lung fibrosis [53]. Exogenous IL-1β induces lung inflammation, alveolar tissue destruction, tissue remodelling, and fibrosis [54], and a high level of IL-18 is detected in IPF [55].

Individuals with pre-existing diseases are more susceptible to SARS-CoV-2 infection and often have negative outcomes [44,56,57]. The inflammasome may induce acute inflammatory responses in patients with pre-existing conditions suffering from chronic inflammation [38]. Patients with non-alcoholic fatty liver disease, liver cirrhosis, or patients that have undergone a liver transplant display higher levels of IL-18 and LDH in the liver and increased levels of caspase-1 in T lymphocytes [38]. Moreover, patients with pre-existing cardiovascular diseases may be vulnerable to COVID-19 [58]. RNA sequencing reveals that patients with non-ischaemic dilated cardiomyopathy or ischaemic cardiomyopathy and patients with COVID-19 share dysregulated immune-associated genes such as ILs and chemokine ligands (CCLs), and NF-κB-associated genes such as *IKBKG* and *NFKBIA* [58]. More importantly, the upregulation of inflammasome-associated genes and inflammatory macrophages are observed in patients with cardiomyopathy and patients with COVID-19 [58]. Moreover, the RNA sequencing of PBMCs from patients with coronary artery diseases displays a similar pattern of immune-associated gene expressions compared to that from patients with COVID-19 [58]. Interestingly, inflammasome-associated genes such as *CHUK* and *NFKBIA* are dysregulated in both patients [58]. These results suggest that immune dysregulation observed in underlying diseases exacerbates COVID-19 severity, and patients with pre-existing diseases are susceptible to severe COVID-19 [38,58].

## 6. Consideration of Targeted Therapies

Many patients with COVID-19 die of immunomodulatory failure and organ damage due to SARS-CoV-2-induced pneumonia, ARDS, or CRS [1,3,4]. Immune-modulating therapies will be required in the COVID-19 pandemic to conquer SARS-CoV-2. Inflammasome-driven caspase-1 activation and IL-1β production after SARS-CoV-2 infection can be suppressed by treatment with MCC950, a selective inhibitor of NLRP3 in primary human monocytes [7]. Furthermore, SARS-CoV-2-induced lytic cell death is reversed by the diabetes medicine glyburide, which abolishes the activation of the NLRP3 inflammasome. Enhanced IL-1β production due to SARS-CoV-2 infection has been shown to be inhibited by treatment with the caspase-1 inhibitor AC-YVAD-CMK or pan-caspase inhibitor Z-VAD-FMK [28]. Metformin, an anti-diabetic, inhibits the SARS-CoV-2-induced pulmonary inflammatory response, which diminishes immune cell recruitment and ASC speck formation in SARS-CoV-2-infected mice [51]. The pan-caspase inhibitor emricasan inhibited the caspase-1 activity of CD4^+^ T lymphocytes in the blood of patients with moderate to severe COVID-19 [29]. Moreover, pre-treatment with dexamethasone or an NF-κB inhibitor BAY-11-7082 suppresses the phosphorylation of NF-κB p65 and IκBα, thereby inhibiting the translocation of NF-κB p65 to the nucleus and the DNA binding of p65 in human PBMCs stimulated by the SARS-CoV-2 S1 protein [9]. The overproduction of IL-1β is attenuated in SARS-CoV-2 S1 protein-stimulated human PBMCs treated with dexamethasone [9]. Although pre-treatment with dexamethasone slightly reduces NLRP3, treatment of CRID3 (NLRP3 inhibitor) efficiently attenuates the increasing levels of NLRP3 and IL-1β as well as the activation of caspase-1 in human PBMCs stimulated by the SARS-CoV-2 S1 protein [9]. In addition, the SARS-CoV-2-mediated activation of caspase-1 and cell lysis are reversed by the IL-1 receptor antagonist (IL-1RA), while AC-YVAD-CMK and IL-1RA suppress levels of pro-inflammatory cytokines IL-6 and TNF-α [28]. Both IL-6 and TNF-α are found in patients with COVID-19 and are responsible for pathological injury [3]. Similarly, treatment with anakinra, which is a recombinant IL-1RA known to reduce pro-inflammatory cytokines such as IL-1α and IL-1β, exhibits beneficial effects on patients with COVID-19 [59]. Treatment with a high-dose of anakinra suppresses hyper-inflammatory symptoms in patients with COVID-19 and exhibits clinical improvements, including that of respiratory function [59]. In addition, the S protein inhibitor EK1 peptide and TMPRSS2 inhibitors such as camostat and nafamostat suppress SARS-CoV-2 entry in LORGs [32]. S protein-induced inflammasome activation is reversed by treatment with MCC950, and the release of IL-1β is also blocked in spite of S-protein exposure. Similarly, treatment with VX-765 (a caspase-1 inhibitor) suppresses NLRP inflammasome activation and the secretion of IL-1β [8]. Additionally, the development of nanovesicles through biomedical engineering may prevent the cytokine storm that occurs in patients with COVID-19 [60]. For instance, 25-hydroxycholesterol and didodecyldimethylammonium bromide (25-HC@DDAB) nanovesicles are lung-specific and with high efficacy [60]. The 25-HC@DDAB inhibits the cytokine storm in PBMCs derived from patients with COVID-19. In addition, the mRNA level of the inflammasome component NLRP3 and the enhanced production of IL-1β are reversed by treatment with 25-HC@DDAB in PBMCs from patients with severe COVID-19 [60]. Based on the reported data, the inflammasome may be a key mediator of the cytokine storm and the modulation of the inflammasome complex may help us understand the pathogenic mechanisms of SARS-CoV-2 and discover new promising therapeutics. Although there are no clinical trials or observations for these potential therapeutic candidates until now, they may be tested and applied in clinical practice. Additionally, it is also important to test any treatments in clinical application for toxicity and efficacy.

## 7. Conclusions

Some patients with COVID-19 display poor clinical outcomes and experience a cytokine storm, which can lead to multiple organ damage and death. The inflammasome appears to contribute to this immune disturbance. The importance of the inflammasome is emerging these days. SARS-CoV-2 can trigger inflammasome activation and the release of pro-inflammatory cytokines (Table 1). Inflammasome components and cytokines can be observed in the plasma or sera from patients with COVID-19 as well as in post-mortem specimens. They appear to have a detrimental effect. However, several in vitro studies have demonstrated that the regulation of the inflammasome has a protective effect against SARS-CoV-2 infection by reducing inflammasome activation, inflammasome-driven cytokine levels, or SARS-CoV-2-induced lytic cell death. Although little is known about the interaction between inflammasome and SARS-CoV-2, it suggests that the inflammasome and its regulation have the potential as therapeutic targets for patients with COVID-19.

## Figures and Tables

**Table 1:** Abnormal inflammasome activation after SARS-CoV-2 infection.

	Materials	Abnormal Inflammasome Activation	References
**Human**	Primary human monocytes	SARS-CoV-2-induced NLRP3 inflammasome activation, caspase-1 activation, IL-1β production, and pyroptosis	[7]
		SARS-CoV-2-driven NLRP3 inflammasome activation, IL-1β production, lytic cell death, and increased LDH levels	[28]
	Human peripheral blood mononuclear cells (PBMCs)	Increased levels of NLRP3 and ASC, and active intracellular caspase-1	[7]
		SARS-CoV-2 S1 protein-induced NF-κB activation, NLRP3 inflammasome, caspase-1 activation, and increased levels of IL-1β	[9]
	Supernatants from PBMCs	High level of IL-1β	[7]
	Human macrophage	SARS-CoV-2 S protein-driven activation of inflammasome and secretion of IL-1β	[8]
	Human serum	High concentration of Casp1p20 and IL-18 in patients with COVID-19	[7]
		Inflammasome-induced increased levels of IL-1β and IL-18 in patients with COVID-19	[31]
	Postmortem lung tissue	High number of NLRP3 and ASC puncta in patients with COVID-19	[7]
		Higher levels of NLRP3, ASC specks, and caspase-1 in leukocytes in the lungs from patients with COVID-19	[50]
	Human hematopoietic stem cells (HSCs)	SARS-CoV-2 S protein-induced activation of NLRP3 inflammasome	[23]
		SARS-CoV-2 S protein-induced NLRP3 inflammasome activation and cytosolic activity of caspase-1	[33]
	Very small embryonic-like stem cells (VSELs)	SARS-CoV-2 S protein-induced activation of NLRP3 inflammasome	[23]
	Endothelial progenitor cells (EPCs)	SARS-CoV-2 S protein-induced NLRP3 inflammasome activation and cytosolic activity of caspase-1	[33]
**Animal**	K18-hACE2 mice	SARS-CoV-2 S1 subunit-triggered NF-κB signalling and IL-1β release	[5]

## Data Availability

Not applicable.
